# Reading Performance and Compensatory Head Posture in Infantile Nystagmus after Null Zone Training

**DOI:** 10.3390/ijerph16234728

**Published:** 2019-11-27

**Authors:** Norliza Mohamad Fadzil, Zainora Mohammed, Mizhanim Mohamad Shahimin, Noor Haziq Saliman

**Affiliations:** Program of Optometry and Vision Science, Faculty of Health Sciences, Universiti Kebangsaan Malaysia Kampus Kuala Lumpur (UKMKL), Jalan Raja Muda Abdul Aziz, Kuala Lumpur 50300, Malaysia; zainora@ukm.edu.my (Z.M.); mizhanim@ukm.edu.my (M.M.S.); haziq.saliman@yahoo.com (N.H.S.)

**Keywords:** null zone, infantile nystagmus, compensatory head posture, reading

## Abstract

This study aimed to assess the visual function, reading performance, and compensatory head posture (CHP) in schoolchildren with infantile nystagmus. A total of 18 participants aged between 13 to 18 years old were divided into spectacle (*n* = 9) and null zone group (*n* = 9) based on their visual acuity. Visual acuity (LogMAR), contrast sensitivity (Pelli–Robson), reading time and rate (Tobii TX300), and CHP were measured pre and post null zone reading training. Participants in the null zone group received 10 sessions of training (5 weeks). Visual acuity and contrast sensitivity of participants in the spectacle and null zone groups were not significantly different pre and post training. Reading performance, i.e., reading time (*z* = −1.36; *p* = 0.173) and reading rate (*z* = −0.06; *p* = 0.953), of participants in the spectacle group was not significantly different after 5 weeks. Reading time (*z* = −2.55; *p* = 0.011) and reading rate (*z* = −2.07; *p* = 0.038 of participants in the null zone group showed significant improvement post training. After 5 weeks, CHP improved in six out of the nine participants (66.7%) of the null zone group and was unchanged in all participants in the spectacle group. Null zone reading training could benefit children with infantile nystagmus in improving reading performance and compensatory head posture.

## 1. Introduction

Vision impairment in children may occur as a result of congenital, hereditary, inflammatory, and infectious processes or trauma. The major causes of vision impairment and blindness in children are influenced by the level of socioeconomic development and availability of health care services [1]. In Malaysia, infantile nystagmus (15%) was reported to be the second after congenital cataract (17%) of major causes of vision impairment in children [2]. It was diagnosed in 0.06% of special school children aged between 7 and 17 years of age in Malaysia [3]. Infantile nystagmus is an involuntary, bilateral, conjugate, and rhythmic oscillation of the eyes present at birth to 6 months of age [4]. Null zone is one of many important features of infantile nystagmus and it may present either in eccentric gaze or in the primary position [5]. Generally, young children will learn to adopt a CHP if the null zone is eccentric and advantageous to improve their visual acuity or minimize oscillation.

Previous studies reported that the poor visual acuity that is often encountered amongst persons with infantile nystagmus is partly due to the involuntary retinal image movement [6]. An abnormal oculomotor control in infantile nystagmus causes the image of a target to move rapidly away from and back to the fovea and thus result in reduced vision and limited visual performance [4]. Previous studies have reported that reading speed in persons with nystagmus is reduced compared to those without vision impairment [7]. When persons with nystagmus perform visually demanding tasks such as reading, the intensity of nystagmus can increase and consequently may reduce the acuity [4].

A majority of persons with infantile nystagmus adopt a compensatory head posture in order to make use of the null zone [5]. If the position of the null zone is in the region of primary position, compensatory head posture may not develop. On the other hand, if the position of a null zone is in an eccentric gaze position, a compensatory head posture is usually developed to perform tasks that require detailed vision as retinal slip is minimized and visual acuity is optimized [8,9]. Abadi and Bjerre (2002) reported that 73% (105 of 143 participants) of infantile nystagmus participants exhibited null zones within plus or minus 10° of the primary position and all of their participants (*n* = 38) with null zones at or beyond plus or minus 20° adopted a constant compensatory head posture [4]. Hertle and Dell’Osso (1999) reported that 60% of persons with infantile nystagmus have a null zone in the eccentric gaze [10]. The children may adopt convergence if null zone is in primary position [11].

It has been reported that infantile nystagmus accounts for 20% of ocular compensatory head postures and it occurs in one in 1000–5000 of the population [12]. In a study in the US, it was reported that the annual incidence of nystagmus in children younger than 19 years old is 6.72 per 100,000 [13]. Maintaining a large angled compensatory head posture in young children whilst performing tasks that require visual effort can be cosmetically unacceptable and may lead to neck pain and muscle contracture. Therefore, management strategies have been designed to shift a null zone as close to the primary position to eliminate the compensatory head posture either for cosmetic reasons or to eliminate later orthopedic problems due to the long-term contracture of the neck muscles [5].

The therapeutic options available for persons with infantile nystagmus such as optical treatment, surgical, pharmaceutical, and electrical stimulation are designed to either induce convergence or to shift the null zone to the primary position, reducing the intensity of the nystagmus and prolonging foveation periods [14,15]. Optical treatment such as correction of the refractive error using spectacles in persons with nystagmus has been reported to increase the aberrations and visual distortion when they look through the peripheral area of the lens [16]. Contact lenses on the other hand, provide a wider field of view and reduced optical distortions compared to spectacles [17]. Although contact lenses are well tolerated with a low risk profile, nystagmus patients fitted with contact lenses must be monitored closely to minimize any potential contact lens complications [18,19]. Surgical treatment to reposition the eye muscles may also be performed in those with significant compensatory head posture [20,21]. However, long term follow-up of surgical treatment for nystagmus may cause under- or over-correction of the head postures and limitations of eye movements post-operatively [20].

Null zone reading strategy is another treatment option available to exploit the null zone and reduce compensatory head posture. This training involves teaching people with nystagmus to place objects of interest in the null zone position so that they turn their eyes into the position of gaze where the nystagmus is minimal or absent. Null zone reading strategy has the advantage that the person may opt to cease using the strategy at any time as it has no irreversible effects. It also does not have associated undesired post-surgical under- or over-corrected head postures and limitation of eye movement [20]. However, the null zone reading strategy has not been widely adopted, and very few studies have been reported in the literature regarding this training [22]. Previous studies have reported improved reading performance following null zone reading training in participants with nystagmus [22,23]. Thus, the aim of this study is to investigate the impact of null zone training on visual functions, reading performance, and compensatory head posture in schoolchildren with infantile nystagmus.

## 2. Materials and Methods

### 2.1. Participants

This study used a convenience sampling method, targeting schoolchildren with infantile nystagmus attending Special Education Schools for the Blind, Setapak, Kuala Lumpur. This study was conducted according to the Helsinki Declaration, and permission to conduct this research was granted by the Ministry of Education Malaysia, the school and Ethics Research Committee, Universiti Kebangsaan Malaysia (UKM1.5.3.5/244/NN-055-2012). Information about the research and a consent form were given to the parents of all children with low vision. Once informed consent was obtained, a standard vision assessment was performed. Participants were children with nystagmus aged 13–18 years old; distance visual acuity (VA) was 6/60 or better; no changes in VA for the last 12 months prior to recruitment; no physical or mental disabilities. Participants were ineligible if they had null zone on convergence and had another ocular pathology. Participants who were not eligible for the research were referred to the Optometry Clinic for annual vision assessment. A total of 18 participants aged between 13 and 18 years of age were recruited and randomly assigned into spectacle (*n* = 9) and null zone group (*n* = 9). The sample size of the participants in this study was calculated using Cochran’s formula (1977) [24] based on nystagmus prevalence, *p*, of 0.6% in Malaysia [3].

(1) $$Sample\;size=\frac{z^{2}p\left(1-p\right)}{\Delta^{2}}=\frac{1.96^{2}\left(0.006\right)\left(1-0.006\right)}{0.05^{2}}=9$$

### 2.2. Procedures

Participants with refractive error were given a pair of glasses. After 2 weeks of wearing spectacles, baseline (pre) measurements were performed, i.e., distance and near visual acuity using the LogMAR chart and MNread chart, respectively, contrast sensitivity using the Pelli–Robson chart, and assessment of compensatory head posture. Reading performance measured was reading time and reading rate using the Tobii TX300 eye tracker by projecting text consisting of 50 words.

The position of the null zone of each participant was determined by the careful study of ocular movements. The participant was instructed to follow the movement of a light whilst maintaining his/her head in primary position. The position in which the eye movement was minimal or diminished was recorded as the null zone.

The change in head posture was determined qualitatively. Photographs of every participant were taken whilst reading a text at baseline and after training to assess CHP. A grid was superimposed on the center of the photo of each participant. A vertical line that passes through the nose bridge and a horizontal line that passes through the pupils were used as the reference points to determine the head posture. Participants who did not meet these criteria were considered to have CHP.

### 2.3. Null Zone Reading Strategy

The null zone reading strategy taught is easy and simple to ensure that the children are able to understand and use it effectively. If the nystagmus was not completely nulled, the position of least nystagmus was used for null zone training. The participant sits with the head in the primary position. Training materials were positioned on a reading stand and placed in the position of the null zone of the participant. The participant was instructed to look at the training material by moving only the eyes. Training material that consisted of letters that can be easily seen by the participant was used initially. Once the participants were confident and comfortable with the technique, the training progressed towards more difficult tasks using words, sentences, and short paragraphs. To read the sentences, the participant was required to move the head across the training material whilst maintaining the eyes at null zone. Each participant received 10 sessions of training within 5 weeks. Previous studies reported that the optimal training sessions for visually impaired persons to help develop their visual skills when reading was between 3 and 10 sessions of approximately one hour each, combined with home practice [25,26]. To determine changes in visual function, reading performance, and compensatory head posture after training, measurements of the parameters were performed immediately post training.

## 3. Results

The comparison of visual function and reading performance at baseline and post training within spectacle and null zone groups was performed using Wilcoxon sign rank test. The change in CHP of participants both in the spectacle and the null zone groups was presented as a percentage.

The parameters of the study, i.e., visual functions, reading performance, and compensatory head posture were compared within groups at baseline and post training (after 5 weeks). Participants in the spectacle group acted as a control in which they only received spectacles as treatment. Participants in the null zone group received ten sessions of null zone training after baseline measurement was performed.

### 3.1. Spectacle Group

The result of the study showed that parameters of visual function (visual acuity and contrast sensitivity) and reading performance (reading time and reading rate) of participants in the spectacle group were not significantly different when compared at baseline and after 5 weeks (Table 1).

Using the grid overlay placed over each photo of the participants, it was estimated that the compensatory head posture was not present in 2 (22.2%) and unchanged in 7 (77.8%) participants after 5 weeks (Table 2).

### 3.2. Null Zone Group

Participants in the null zone group showed significant improvement in reading time (median reading time _baseline_ = 95.93 words/min; median reading time _post train_ = 90.75 min, z = −2.55; *p* = 0.011) and reading rate (median reading rate _baseline_ = 31.27 min; median reading rate _post train_ = 31.30 words/min *z* = −2.07; *p* = 0.038), i.e., an increment of approximately 20% post training (Table 1). However, there was no significant change in visual acuity (near and distance) and contrast sensitivity post training (Table 1).

It was estimated that the compensatory head posture of participants in the null zone group was not present in two participants (22.2%), improved in six participants (66.7%), and remained unchanged in one (11.1%) participant post training (Table 2).

## 4. Discussion

Visual function parameters, i.e., visual acuity (near and distance) and contrast sensitivity, both in the spectacle and the null zone group did not show significant improvement statistically after 5 weeks of training. The improvement of the near visual acuity in both groups was equivalent to one line. Although this value was not statistically significant, it was considered to be clinically significant [27]. The finding indicates that prescribing spectacle correction to children with infantile nystagmus assist them in having better visual acuity. This is essential as individuals with infantile nystagmus usually have better near than distance vision due to the dampening of nystagmus on convergence [12]. Thus, providing the required spectacle correction will assist schoolchildren with reading-related tasks at school. This is important as visual acuity and refractive error have been reported to be associated with academic performance in children [28].

Reading performance (reading time and reading speed) of participants in the null zone group improved significantly post training. This outcome suggests that the null zone training had an effect on the reading performance. When using the null zone reading strategy, the child’s eyes were placed in the position of minimal or diminished nystagmus, which helps facilitate the achievement of best possible vision. This finding thus supports the literature reporting that the use of the null zone improves reading efficiency [22]. However, the impact of practice may also have influence in the improvement of reading performance. All participants in this study were school children in whom reading is a daily activity during the learning session. These children are likely to have continued to practice using the null zone reading strategy during their school hours and also during their revision after training had been concluded [29]. Mastering the null zone reading strategy and practice assists in increasing the reading performance. Almost half of all academic related tasks in school are conducted at near distances, and on average, children engaged in continuous reading for 23 ± 5 min at a time [28]. Thus, improving reading performance in schoolchildren with nystagmus is of priority.

In this study, 6 out of 7 participants in the null zone group who have CHP showed an improvement towards primary gaze after training was given. However, CHP was unchanged in six participants of the spectacle group. A previous study reported that continued use of CHP leads to permanent contracture of the neck muscle and results in inappropriate posture [12]. In this study, the null zone reading strategy provides the benefits of using a null zone whilst reducing or eliminating the need for a CHP, thus removing the unwanted complications. The eyes were placed in the null zone position by relocating the fixation object instead of using prisms or a compensatory head posture. The advantage of the null zone reading strategy is that it does not have the associated limitations and undesired post-surgical results. Previous studies have shown that at long term follow ups, surgical treatment for nystagmus may cause under- or over-correction of the head postures and limitations of eye movements post-operatively [20], and prisms have effects such as reduced image clarity and increasing the weight of glasses [5].

## 5. Conclusions

The null zone reading strategy is an alternative to surgery to improve reading performance and reducing compensatory head posture, especially for schoolchildren.

## Figures and Tables

**Table 1:** Comparison of visual function and reading performance at baseline and post training within spectacle and null zone groups using Wilcoxon sign rank test.

Variables	Baseline (Spectacle) *n* = 9 Median	Post Training (Spectacle) *n* = 9 Median	Baseline (Null Zone) *n* = 9 Median	Post Training (Null Zone) *n* = 9 Median
**Best Corrected VA (Distance)** **LogMAR**	0.80	0.80	0.70	0.70
	*Z* = −1.41; *p* = 0.157		*z* = −1.73; *p* = 0.083	
**Best Corrected VA (Near)** **LogMAR**	0.90	0.70	0.80	0.70
	*z* = −1.86; *p* = 0.063		*z* = −1.84; *p* = 0.066	
**Reading Time** **(min)**	90.30	74.09	95.93	90.75
	*z* = −1.36; *p* = 0.173		*z* = −2.55; *p* = 0.011	
**Reading Rate** **(words/min)**	32.56	38.87	31.27	31.30
	*z* = −0.06; *p* = 0.953		*z* = −2.07; *p* = 0.038	
**Contrast Sensitivity**	1.35	1.65	1.50	1.65
	*z* = −1.00; *p* = 0.317		*z* = −1.414; *p* = 0.157	

**Table 2:** Summary of the presence, type, and change in CHP in spectacle and null zone groups.

Subject	Spectacle Group (*n* = 9)			Null Zone Group (*n* = 9)		
	Presence of CHP	Type of CHP Baseline	Change in CHP Post Training	Presence of CHP	Type of CHP Baseline	Change in CHP Post Training
1	No	No CHP	No CHP	No	No CHP	No CHP
2	No	No CHP	No CHP	No	No CHP	No CHP
3	Yes	Head tilt left	Unchanged	Yes	Head tilt left	Yes/Reduce
4	Yes	Head tilt left	Unchanged	Yes	Head tilt left	Yes/Reduce
5	Yes	Head tilt left	Unchanged	Yes	Head tilt left	Yes/Reduce
6	Yes	Head tilt right	Unchanged	Yes	Face turn left	Yes/Reduce
7	Yes	Head tilt right	Unchanged	Yes	Face turn right	Yes/Reduce
8	Yes	Face turn right	Unchanged	Yes	Head tilt right	Unchanged
9	Yes	Face turn left	Unchanged	Yes	Face turn right	Yes/Reduce
